# Associations between Biomarkers of Exposure and Lung Cancer Risk among Exclusive Cigarette Smokers in the Golestan Cohort Study

**DOI:** 10.3390/ijerph18147349

**Published:** 2021-07-09

**Authors:** Brian L. Rostron, Jia Wang, Arash Etemadi, Sapna Thakur, Joanne T. Chang, Deepak Bhandari, Julianne Cook Botelho, Víctor R. De Jesús, Jun Feng, Mitchell H. Gail, Maki Inoue-Choi, Reza Malekzadeh, Akram Pourshams, Hossein Poustchi, Gholamreza Roshandel, Meredith S. Shiels, Qian Wang, Yuesong Wang, Baoyun Xia, Paolo Boffetta, Paul Brennan, Christian C. Abnet, Antonia M. Calafat, Lanqing Wang, Benjamin C. Blount, Neal D. Freedman, Cindy M. Chang

**Affiliations:** 1 Center for Tobacco Products, Food and Drug Administration, Silver Spring, MD 20993, USA; jia.wang@fda.hhs.gov (J.W.); sapna.thakur@fda.hhs.gov (S.T.); joanne.chang@fda.hhs.gov (J.T.C.); cindy.chang@fda.hhs.gov (C.M.C.); 2 Metabolic Epidemiology Branch, Division of Cancer Epidemiology and Genetics, National Cancer Institute, National Institutes of Health, Bethesda, MD 20892, USA; arash.etemadi@nih.gov (A.E.); maki.inoue-choi@nih.gov (M.I.-C.); abnetc@mail.nih.gov (C.C.A.); freedmanne@mail.nih.gov (N.D.F.); 3 Digestive Oncology Research Center, Digestive Diseases Research Institute, Tehran University of Medical Sciences, Tehran 1411713135, Iran; dr.reza.malekzadeh@gmail.com (R.M.); akrampourshams@gmail.com (A.P.); 4 Division of Laboratory Sciences, Centers for Disease Control and Prevention, Atlanta, GA 30341, USA; xwo1@cdc.gov (D.B.); gur5@cdc.gov (J.C.B.); FOA5@cdc.gov (V.R.D.J.); czf2@cdc.gov (J.F.); uxi5@cdc.gov (Y.W.); Vvq2@cdc.gov (B.X.); aic7@cdc.gov (A.M.C.); lfw3@cdc.gov (L.W.); bkb3@cdc.gov (B.C.B.); 5 Biostatistics Branch, Division of Cancer Epidemiology and Genetics, National Cancer Institute, National Institutes of Health, Bethesda, MD 20892, USA; gailm@exchange.nih.gov; 6 Liver and Pancreaticobilliary Research Center, Digestive Diseases Research Institute, Tehran University of Medical Sciences, Tehran 1411713135, Iran; hossein.poustchi@gmail.com; 7 Golestan Research Center of Gastroenterology and Hepatology, Golestan University of Medical Sciences, Gorgan 4917867439, Iran; roshandel_md@yahoo.com; 8 Infections and Immunoepidemiology Branch, Division of Cancer Epidemiology and Genetics, National Cancer Institute, Bethesda, MD 20892, USA; shielsms@mail.nih.gov; 9 Division of Hematology and Medical Oncology, Icahn School of Medicine at Mount Sinai, New York, NY 10029, USA; Qian.Wang2@mountsinai.org; 10 Stony Brook Cancer Center, Stony Brook University, Stony Brook, NY 11794, USA; paolo.boffetta@mssm.edu; 11 Department of Medical and Surgical Sciences, University of Bologna, 40138 Bologna, Italy; 12 International Agency for Research on Cancer, 69372 Lyon, France; Brennan@iarc.fr

**Keywords:** tobacco, biomarker, lung cancer

## Abstract

Biomarkers of tobacco exposure are known to be associated with disease risk but previous studies are limited in number and restricted to certain regions. We conducted a nested case–control study examining baseline levels and subsequent lung cancer incidence among current male exclusive cigarette smokers in the Golestan Cohort Study in Iran. We calculated geometric mean biomarker concentrations for 28 matched cases and 52 controls for the correlation of biomarker levels among controls and for adjusted odds’ ratios (ORs) for lung cancer incidence by biomarker concentration, accounting for demographic characteristics, smoking quantity and duration, and opium use. Lung cancer cases had higher average levels of most biomarkers including total nicotine equivalents (TNE-2), 4-(methylnitrosamino)-1-(3-pyridyl)-1-butanol (NNAL), and 3-hydroxyfluorene (3-FLU). Many biomarkers correlated highly with one another including TNE-2 with NNAL and N-Acetyl-S-(2-cyanoethyl)-L-cysteine (2CYEMA), and N-Acetyl-S-(4-hydroxy-2-buten-1-yl)-L-cysteine (t4HBEMA) with N-Acetyl-S-(3-hydroxypropyl-1-methyl)-L-cysteine (3HMPMA) and N-Acetyl-S-(4-hydroxy-2-methyl-2-buten-1-yl)-L-cysteine (4HMBEMA). Lung cancer risk increased with concentration for several biomarkers, including TNE-2 (OR = 2.22, 95% CI = 1.03, 4.78) and NNN (OR = 2.44, 95% CI = 1.13, 5.27), and estimates were significant after further adjustment for demographic and smoking characteristics for 2CYEMA (OR = 2.17, 95% CI = 1.03, 4.55), N-Acetyl-S-(2-carbamoylethyl)-L-cysteine (2CAEMA) (OR = 2.14, 95% CI = 1.01, 4.55), and N-Acetyl-S-(2-hydroxypropyl)-L-cysteine (2HPMA) (OR = 2.85, 95% CI = 1.04, 7.81). Estimates were not significant with adjustment for opium use. Concentrations of many biomarkers were higher at the baseline for participants who subsequently developed lung cancer than among the matched controls. Odds of lung cancer were higher for several biomarkers including with adjustment for smoking exposure for some but not with adjustment for opium use.

## 1. Introduction

It is well established that cigarette smoking causes lung cancer [1,2,3] and many constituents of cigarette tobacco and smoke have been identified as carcinogens or potential carcinogens [4,5]. For example, the tobacco-specific nitrosamines (TSNAs) 4-(methylnitrosamino)-1-(3-pyridyl)-1-butanone (NNK) and its metabolite, 4-(methylnitrosamino)-1-(3-pyridyl)-1-butanol (NNAL), are powerful lung carcinogens [5], and N’-nitrosonornicotine (NNN) causes oral cancer [6]. Other carcinogens found in cigarette smoke include polycyclic aromatic hydrocarbons (PAHs) such as benzo[*a*]pyrene and volatile organic compounds (VOCs) such as benzene and formaldehyde [4,7].

Several prospective cohort studies have been used to examine the relationship between tobacco smoke constituents and cancer risk using biomarker of exposure data. For example, Church et al. [8] conducted a nested case–control study of cigarette smokers using data from the Prostate, Lung, Colorectal, and Ovarian Cancer Screening Trial (PLCO) in the U.S. They found that baseline serum NNAL was significantly associated with subsequent incidence of lung cancer even after adjustment for demographic characteristics, smoking duration, and levels of cotinine, the principal metabolite of nicotine, and r-1,t-2,3,c-4-tetrahydroxy-1,2,3,4-tetrahydrophenanthrene (PheT), one PAH metabolite. Researchers have also analyzed baseline biomarker data for subsequent smoking cancer cases and controls in the Shanghai Cohort Study [9]. They found that cotinine, NNAL, and PheT were significantly associated with lung cancer risk even after adjusting for the number of cigarettes smoked per day (CPD), number of years of smoking, and levels of the other two biomarkers [10]. They also found a similar relationship between NNN and esophageal cancer [11]. They did not find an association between lung cancer risk and the metabolites of five VOCs, including acrolein and benzene, after adjustment for cotinine levels [12]. Another analysis of the Shanghai Cohort Study and Singapore Chinese Health Study also found associations between cotinine, NNAL levels, and lung cancer incidence, adjusting for smoking history and levels of the other biomarker [13]. A related prospective study among people who have never smoked in their lifetime in the Shanghai cohort focused on three PAHs including PheT and metabolites of four VOCs [14]. This study also found associations with lung cancer risk for the PAHs but not the VOCs. The use of biomarker data in these studies, in addition to self-reported behavioral information, provided quantitative measures of exposure for cigarette smoke and its constituents. 

This study examines urinary biomarker levels and lung cancer risk utilizing the resources provided by the Golestan Cohort Study (GCS), a population-based prospective cohort study conducted in the Golestan Province of northeastern Iran [15]. Prior analyses of this cohort have examined the use of tobacco products such as cigarettes, waterpipe, and nass (a type of smokeless tobacco used in the region that also includes ash and lime) among study participants and analyzed data on biomarkers of exposure from users of these products [16,17]. In the current analysis, we examine associations between baseline concentrations of nicotine, TSNAs, and PAHs and VOC metabolites, and the subsequent lung cancer incidence among exclusive cigarette smokers. In doing so, we provide additional information about these associations using a cohort study from a region of the world in which these relationships have not been previously studied.

## 2. Materials and Methods

Slightly more than 50,000 residents of the Golestan Province aged 40 to 75 years were recruited for the GCS between 2004 and 2008 [15]. Approximately 20% of the participants lived in the city of Gonbad Kavus and the remainder consisted of eligible residents from 326 rural villages. The primary purpose of the study was to investigate the relationship between risk factors and chronic disease in this population, in particular esophageal cancer [15]. Study participants were interviewed by a trained interviewer using a structured questionnaire. Participants provided information on topics such as demographic characteristics, socioeconomic status, and medical history, as well as use of tobacco products, alcohol, and opium in the study’s general questionnaire [18]. Participants were asked about their use of cigarettes, nass, and waterpipes and pipes, including frequency, the amount of use, and ages at which they began and ended their use. This study used information reported at baseline. Participants were also asked to provide baseline biospecimens including a spot urine sample at the time of recruitment. The GCS and its study protocols were approved by the institutional review boards of Tehran University of Medical Sciences, the International Agency for Research on Cancer (IARC), and the National Cancer Institute [15]. The involvement of the Centers for Disease Control and Prevention (CDC) laboratories did not constitute engagement in human subject research.

GCS participants were monitored after the study enrollment through annual telephone interviews and home visits through the end of 2017 [18]. In the event of a participant’s cancer diagnosis or death, a study staff member was sent to the home of the individual to collect detailed information and the participant’s medical records were obtained from their medical center. The collected information was independently evaluated by at least two physicians to produce a cancer determination based on the 10th Revision of the International Statistical Classification of Diseases and Related Health Problems (lung cancer: C34). All cancer diagnoses were further confirmed through linkage to the Golestan Cancer Registry which is included in the IARC’s Cancer Incidence in Five Continents series [19].

This analysis used the resources provided by the GCS to perform a nested case–control study examining the relationship between biomarkers of exposure and lung cancer risk. Up until 1 January 2018, a total of 116 probable cases of lung cancer accrued in the cohort. We used risk-set sampling to randomly select two controls for each case, matched by tobacco use, age, sex, place of residence (urban, living in Gonbad Kavus/rural, living in a village), enrollment period, and duration of the follow-up in the cohort. For the duration of the follow-up, the cases were matched with the controls from the same enrollment period who did not have lung cancer at the time of lung cancer diagnosis. The analysis was limited to current exclusive cigarette smokers who reported daily or non-daily smoking, smoked at least once a week for at least six months, and never used a waterpipe tobacco or nass at the time of enrollment. Participants could not have been diagnosed with lung cancer and were free of other cancers at baseline. A previous study of mortality risks among tobacco users in the GCS found that the exclusion of cases occurring during the first two years of the follow-up did not affect estimates [20]. A total of 31 confirmed cases and 58 controls were identified from this group. Three study participants with extremes in hydration (i.e., urinary creatinine levels outside the range of 10–370 mg/dL) were excluded from the analysis [21], as were six participants who were no longer matched to an eligible case or control, resulting in the inclusion of 28 cases and 52 controls in the analysis. Figure S1 in the Supplementary Material graphically presents the selection of participants. The mean follow-up time between the biospecimen collection and cancer diagnosis for the cases was 5.1 years with a range of 0 to 11 years.

The measurement of biomarkers in the baseline urine samples was conducted at the Division of Laboratory Sciences of the National Center for Environmental Health at the CDC and detailed information about the assay methodology has been presented previously [16]. Panels consisted of tobacco alkaloids (7 nicotine metabolites and 2 minor alkaloids), TSNAs (4 compounds), PAH metabolites (7 compounds), and VOC metabolites (19 compounds). The results for all measured biomarkers are presented as supplementary material (Tables S1–S3) and a group of biomarkers were selected for presentation in the main body as representatives of different classes of harmful or potentially harmful constituents because of their health impact. For example, the TSNAs NNAL and NNN were selected as powerful carcinogens [5,6]. Metabolites of the VOCs acrylonitrile and 1,3-butadiene were selected due to their substantial cancer risks and a metabolite of acrolein was selected due to its respiratory effects [22]. Of all the biomarkers evaluated in this study, we present results for two TSNAs (NNAL and NNN), two PAH metabolites (1-hydroxypyrene (1-PYR) and 3-hydroxyfluorene (3-FLU)), and ten VOC metabolites (N-acetyl-S-(2-carbamoylethyl)-L-cysteine (2CAEMA): a biomarker for acrylamide; N-acetyl-S-(2-cyanoethyl)-L-cysteine (2CYEMA)-acrylonitrile; N-acetyl-S-(2-carboxyethyl)-L-cysteine (2COEMA) and N-acetyl-S-(3-hydroxypropyl)-L-cysteine (3HPMA)-acrolein; mandelic acid (MADA)-styrene; phenylglyoxylic acid (PHGA)-ethylbenzene and styrene; N-acetyl-S-(2-hydroxypropyl)-L-cysteine (2HPMA)-propylene oxide; N-acetyl-S-(4-hydroxy-2-buten-1-yl)-L-cysteine (t4HBEMA)-1,3-butadiene; N-acetyl-S-(3-hydroxypropyl-1-methyl)-L-cysteine (3HMPMA)-crotonaldehyde; and N-acetyl-S-(4-hydroxy-2-methyl-2-buten-1-yl)-L-cysteine (4HMBEMA)-isoprene). Nicotine itself is not the main cause of cancer from tobacco use [23] but nicotine and its metabolites such as cotinine are often used as measures of smoking exposure [24]. As such, total nicotine equivalents (TNE-2, the molar sum of cotinine, and trans-3′-hydroxycotinine) were used as a measure of overall nicotine exposure in this analysis [25].

Ever regular use of a substance including cigarettes, opium, and alcohol was defined as having ever used at least once a week for at least six months. Socioeconomic status was assessed using quartiles of a previously developed wealth score based on characteristics such as occupation and ownership of property, vehicles, and appliances [26]. Other control variables included education (none, 1–8 years, and 9+ years), ethnicity (Turkmen and non-Turkmen), regular opium use (never used and ever used), regular alcohol use (never used and ever used), and body mass index based on measured height and weight (<25, 25–29, 30+ kg/m^2^). Opium is used in various forms in the region and can be ingested, injected, or smoked [18].

The distribution of demographic and substance use characteristics, mean age at smoking initiation, mean CPD, and geometric mean biomarker concentrations were calculated for the cases and controls. Biomarker concentrations were adjusted by dividing by urinary creatinine as a measure of hydration. The means and 95% confidence intervals of the log-transformed values were calculated and exponentiated to obtain geometric means and confidence intervals. Biomarker levels below the limit of detection were replaced by the limit of detection divided by the square root of 2 [27]. For most biomarkers, fewer than 3 participants had values lower than the limit of detection with the exception of 18 participants for NNN and 9 for PHGA. Four participants did not have information for NNN and NNAL. *P*-values were calculated using two-sided chi-squared tests for differences of frequencies and two-sided *t*-tests for differences of means. Values less than 0.05 were considered statistically significant. Multiple comparisons of biomarker concentrations were controlled for with Benjamini and Hochberg’s False Discovery Rate procedure [28]. An adjusted *p*-value, 0.05 * (the unadjusted *p*-value rank/the total number of biomarker comparisons), was calculated and the null hypothesis of no difference between log-transformed means was rejected if the unadjusted *p*-value was less than or equal to the adjusted *p*-value. The correlation between biomarker values for controls was calculated as the Pearson correlation coefficients of creatinine-corrected and log-transformed biomarker values. Associations between log-transformed biomarker concentrations and lung cancer incidence were analyzed using conditional logistic regression analysis. This analysis involved accounting for the matching and adjusting for the log-transformed creatinine values as a covariable and then sequentially adjusting for the demographic characteristics of education and ethnicity; demographic characteristics and the regular use of opium; demographic characteristics, CPD, and years of cigarette smoking; and demographics characteristics, opium use, smoking quantity, and duration. The results represent the change in lung cancer risk associated with one log-unit change of the relevant biomarker.

## 3. Results

Table 1 presents demographic and substance use characteristics of study participants. All participants were male and mostly from rural areas. Most participants were middle-aged with 79% being between 40 and 59 years of age at enrollment. Among the cases, 68% regularly used opium, whereas only 33% of the controls had. The cases smoked on average 16.6 CPD and controls 12.6 CPD.

Table 2 presents geometric mean biomarker concentrations for participants. The cases had higher levels of all but two of the biomarkers. For example, mean TNE-2 was 57.3 nmol/mg creatinine (95% CI = 46.0, 71.3 nmol/mg) among the cases and 13.5 nmol/mg (95% CI = 6.9, 26.3 nmol/mg) among the controls. Mean NNAL was 0.280 ng/mg (95% CI = 0.213, 0.368 ng/mg) among the cases and 0.140 ng/mg (95% CI = 0.099, 0.199 ng/mg) among the controls, and mean 3-FLU was 1.88 ng/mg (95% CI = 1.45, 2.44 ng/mg) among the cases and 1.04 ng/mg (0.73, 1.48 ng/mg) among the controls.

Table 3 presents the correlation between biomarkers among the controls. All correlation coefficients were statistically significant with the exception of those for 2CAEMA with NNN and 4HMBEMA and all those involving PHGA. Many of the biomarkers measured were highly correlated with each other, with particularly high correlation coefficients for TNE-2 with 2CYEMA at 0.926 and NNAL at 0.907. Levels of several of the VOC metabolites including 3HPMA, t4HBEMA, 3HMPMA, and 4HMBEMA were also highly correlated, with the correlation coefficients for t4HBEMA being 0.943 for 3HMPMA and 0.919 for 4HMBEMA.

Table 4 presents results from the conditional logistic regression analysis of lung cancer incidence by biomarker concentration. The risk of lung cancer increased significantly with each log-unit change of TNE-2 (OR = 2.22, 95% CI = 1.03, 4.78), NNN (OR = 2.44, 95% CI = 1.13, 5.27), 2CAEMA (OR = 2.00, 95% CI = 1.03, 3.88), 2CYEMA (OR = 2.17, 95% CI = 1.03, 4.58), 3HPMA (OR = 2.19, 95% CI = 1.03, 4.66), MADA (OR = 3.63, 95% CI = 1.00, 13.10), and 2HPMA (OR = 2.72, 95% CI = 1.16, 6.36), and these estimates were significant with additional adjustment for demographic characteristics for these biomarkers and 1-PYR (OR = 2.47, 95% CI = 1.06, 5.77). Estimates were also significant for 2CAEMA (OR = 2.14, 95% CI = 1.01, 4.55), 2CYEMA (OR = 2.17, 95% CI = 1.03, 4.55), and 2HPMA (OR = 2.85, 95% CI = 1.04, 7.81) with additional adjustment for smoking quantity and duration. None of the estimates were significant with adjustment for demographic characteristics and occurrence of opium use, or adjustment for demographics, opium use, and smoking exposure.

## 4. Discussion

In this study of male exclusive cigarette smokers in Iran, the majority of whom were from rural areas, concentrations of most tobacco-related biomarkers including TNE-2, NNAL, and 3-FLU were higher among the lung cancer cases than controls. Many of the biomarkers were highly correlated with one another, including TNE-2 with NNAL and 2CYEMA and t4HBEMA with 3HMPMA and 4HMBEMA, as nicotine NNAL, and the VOC parent compounds are found in substantial quantities in cigarette smoke [29]. Odds of lung cancer increased with higher concentrations of several biomarkers including TNE-2, NNN, 2CYEMA, and 3HPMA, and estimates for the VOC metabolites, 2CYEMA, 2COEMA, and 2HPMA, were statistically significant after further adjustment for demographic characteristics, smoking quantity, and duration. None of the estimates were significant when the occurrence of regular opium use was adjusted for in the regression model.

This analysis provides additional evidence and information concerning the relationship between tobacco biomarker concentrations and lung cancer incidence, using a cohort study in the Middle East, notably an area that has not been previously studied for this purpose. Previous studies utilized data from the PLCO, Shanghai, and Singapore cohorts [8,9,12,13,14] and found that TSNAs such as NNAL and PAH metabolites such as PheT were associated with increased lung cancer risk among smokers. The results in the current study are generally consistent with these previous findings in that smoker lung cancer cases had higher geometric mean concentrations of NNAL and the PAH metabolites 1-PYR and 3-FLU than the controls, and these results thus provide additional evidence from a cohort study in a population with different ethnic and genetic backgrounds. This study has also found associations between VOC metabolites such as 2CYEMA and 2HPMA and lung cancer incidence even after adjustment for smoking exposure.

There are some differences between these results and those of the previous studies, especially for the TSNAs and PAH metabolites. Some of these biomarkers have previously been found to be associated with lung cancer risk even after adjusting for smoking history and levels of other biomarkers. For example, previous studies have found significant associations between NNAL and lung cancer risk among smokers in the PLCO cohort [8], the Shanghai cohort [10], and the Singapore cohort [13] after controlling for smoking. The estimated mean NNAL concentration for the cases in this study was double that of the controls’. In the conditional logistic regression analysis, the matched odds ratio for the NNAL concentration was 1.46, the odds ratio adjusted for demographic characteristics was 1.44, and the odds ratio adjusted for demographics and smoking exposure was 1.35, although these results were not statistically significant. These observed differences may be partially due to the limited sample size in this analysis. This study included 28 lung cancer cases and 52 controls, whereas there were 100 cases and controls in the PLCO analysis [8], 476 cases and controls in the Shanghai cohort analysis [10], and 91 cases and 93 controls in the Singapore cohort analysis [13]. In contrast to the result for NNAL, the odds ratio for NNN was significant in the matched analysis, although not in the matched analyses that further adjusted for factors including smoking exposure. A previous study of the Shanghai cohort found an association between NNN and esophageal cancer risk [11] but this result for NNN and lung cancer is somewhat unexpected. Previous users of nass were excluded from the analysis and the NNN in smokeless tobacco has been identified as a powerful carcinogen, particularly in the oral cavity [6]. Any potential association between NNN and lung cancer risk could be further investigated.

One particularly important aspect of these results is the prevalence of regular opium use especially among the cases and the lack of significant results in the regression analyses adjusted for opium use. Opium use has been found to be associated with the incidence of lung cancer and cancer overall in the Golestan study [18] and has been classified as a lung carcinogen by the IARC [30]. A previous study regarding biomarkers of exposure among opiate and tobacco users in the GCS found that dual users of opiates and tobacco had higher concentrations of 39 biomarkers including those for TSNAs, PAHs, and VOCs than either exclusive smokers or exclusive opiate users [17]. The analysis also found that opiate use contributed a larger portion of PAH concentrations than the nicotine dose did. Sample sizes were limited to perform separate analyses for opium users and non-users in this study. However, a previous study of GCS smokers found that hazard ratio estimates for overall and cancer mortality were slightly higher among opium users compared to non-users [20]. The presence of another exposure (opium use) that contributes to the biomarker concentrations and lung cancer risk complicates and potentially confounds any associations between smoking and lung cancer in this study. Further studies could examine the relative contributions of opium and tobacco use to lung cancer risk among dual users and explore potential interactive effects between these two exposures.

Other characteristics of the present study’s population could also account for some of the differences between these results and those of previous studies. Particular characteristics of nicotine and other biomarker metabolism among study participants could affect results given that certain genetic alleles have been linked to nicotine metabolism and lung cancer risk in regions such as China and Japan [31]. NNAL concentrations have been found to be lower among cigarette smokers in the GCS than among U.S. smokers in the National Health and Nutrition Examination Survey [16]. Such differences between this group and other populations could also result from the cigarette types used or their smoking histories. Large quantities of cigarettes in Iran are imported or smuggled from other countries [32] and the nicotine content in domestic and imported cigarettes has been found to be similar as measured by high performance liquid chromatography [33]. Both the cases and controls reported beginning to smoke on average in their late 20s in this group, whereas most smoking initiation in the U.S. occurs in the teens or early 20s [34].

Associations between biomarkers of exposure and lung cancer risk, controlling for smoking exposure, could have various interpretations. They could suggest that variations in concentrations of specific biomarkers have particular effects on cancer incidence even with adjustment for the effects of smoking generally. They could also suggest that there is a misclassification of the smoking status or there is residual confounding due to insufficient characterization of smoking exposure, or that sources other than smoking contribute to the biomarker concentrations.

This study has certain limitations. As noted, the sample size of lung cancer cases and thus matched controls was limited, reducing power to detect statistically significant results. Many of the biomarkers in the same classes such as TSNAs and PAH and VOC metabolites were highly correlated with one another, resulting in a difficulty to isolate and identify the portion of increased lung cancer risk that was due to particular biomarkers. Biospecimens were collected at baseline and biomarker concentrations may not reflect the total exposure to constituents over time. A subset of GCS participants provided a follow-up urine sample after an average of five years and biomarker concentrations among continuing smokers were highly correlated [16]. Finally, all of the participants in this analysis were male given that smoking is predominantly practiced by males in the region of study.

Future research could expand on this analysis in this and other cohorts, ideally with a larger sample size. Research of this nature on the association between biomarkers of tobacco exposure and disease risk is informative for public health organizations and government agencies as it provides information on the contribution of specific constituents and toxicants regarding the health effects of tobacco products. Such results can thus be used to inform tobacco control efforts as well as government policies and regulations concerning tobacco products.

## 5. Conclusions

Levels of several biomarkers were higher in lung cancer cases than among the controls in a study of male exclusive cigarette smokers in the Golestan Cohort Study in Iran. Some of these biomarkers were associated with lung cancer risk after adjusting for smoking duration and quantity but not after adjustment for opium use.

## Figures and Tables

**Table 1:** Demographic and substance use characteristics of current exclusive cigarette smoking lung cancer cases and controls in the Golestan Cohort Study.

	Cases (*n* = 28)	Controls (*n* = 52)	*p*-Value
	# (%)		
Sex			
Males	28 (100)	52 (100)	
Residence			
Urban	7 (25)	12 (23)	
Rural	21 (75)	40 (77)	0.847
Age (years)			
40–49	11 (39)	20 (38)	
50–59	11 (39)	21 (40)	
60+	6 (21)	11 (21)	0.995
Education			
None	15 (54)	29 (56)	
1–8 years	11 (39)	20 (38)	
9+ years	2 (7)	3 (6)	1.000
Ethnicity			
Turkmen	18 (64)	39 (75)	
Non-Turkmen	10 (36)	13 (25)	0.313
Wealth score			
1st quartile (lowest)	11 (39)	15 (29)	
2nd quartile	2 (7)	9 (17)	
3rd quartile	7 (25)	13 (25)	
4th quartile (highest)	8 (29)	15 (29)	0.577
Body mass index (kg/m^2^)			
<25	18 (64)	32 (62)	
25–29	7 (25)	16 (31)	
30+	3 (11)	4 (8)	0.766
Opium regular use			
Never used	9 (32)	35 (67)	
Ever used	19 (68)	17 (33)	0.003
Alcohol regular use			
Never used	18 (64)	39 (75)	
Ever used	10 (36)	13 (25)	0.313
Mean age at first cigarette use (SD)	25.9 (12.3)	29.5 (12.9)	0.233
Mean number of cigarettes smoked per day (SD)	16.6 (7.0)	12.6 (9.2)	0.045

**Table 2:** Geometric mean biomarker concentrations for lung cancer cases and controls in the Golestan Cohort Study.

	Cases (*n* = 28)	Controls (*n* = 52)	*p*-Value	Adjusted *p*-Value (2)	False Discovery Rate < 0.05 (3)
	Geometric Mean (95% CI) (1)	Geometric Mean (95% CI)			
TNE-2	57.3 (46.0, 71.3)	13.5 (6.9, 26.3)	0.000	0.006	yes
NNAL	0.280 (0.213, 0.368)	0.140 (0.099, 0.199)	0.002	0.025	yes
NNN	0.012 (0.009, 0.015)	0.005 (0.004, 0.007)	0.000	0.009	yes
3-FLU	1.88 (1.45, 2.44)	1.04 (0.73, 1.48)	0.008	0.031	yes
1-PYR	1.05 (0.81, 1.36)	0.66 (0.52, 0.84)	0.016	0.038	yes
2CAEMA	223.6 (166.0, 301.3)	148.0 (113.5, 193.1)	0.053	0.044	no
2CYEMA	171.2 (138.1, 212.3)	55.6 (34.8, 88.7)	0.000	0.003	yes
2COEMA	272.3 (223.8, 331.4)	195.4 (161.5, 236.5)	0.027	0.041	yes
3HPMA	1426.9 (1103.5, 1845.1)	765.7 (610.3, 960.7)	0.001	0.016	yes
MADA	534.7 (437.7, 653.2)	349.6 (298.1, 409.9)	0.002	0.022	yes
PHGA	113.8 (72.8, 177.6)	93.2 (70.1, 124.0)	0.430	0.047	no
2HPMA	74.0 (61.3, 89.4)	43.7 (35.5, 53.7)	0.000	0.013	yes
t4HBEMA	38.0 (30.3, 47.7)	21.6 (16.8, 27.7)	0.001	0.019	yes
3HMPMA	2189.6 (1718.4, 2790.1)	1271.3 (1002.7, 1611.9)	0.004	0.028	yes
4HMBEMA	41.5 (29.8, 57.6)	21.9 (15.8, 30.4)	0.014	0.034	yes

Abbreviation: CI = confidence interval. (1) Biomarker concentrations are expressed as ng/mg creatinine with the exception of TNE-2 which is given as nmol/mg creatinine. (2) Adjusted *p*-value calculated as α × k/m where α is the significance level (0.05), k is the unadjusted *p*-value rank, and m is the total number of biomarker comparisons tested (15). (3) The null hypothesis was rejected for 2COEMA as the lowest-ranked biomarker for which the *p*-value ≤ adjusted *p*-value and for all higher-ranked biomarkers.

**Table 3:** Pearson correlation of log biomarker levels among lung cancer controls (*n* = 52) in the Golestan Cohort Study.

	**TNE-2**	**NNAL**	**NNN**	**3-FLU**	**1-PYR**	**2CAEMA**	**2CYEMA**	**2COEMA**	**3HPMA**	**MADA**	**PHGA**	**2HPMA**	**t4HBEMA**	**3HMPMA**	**4HMBEMA**
TNE-2	1.000														
NNAL	0.907	1.000													
NNN	0.704	0.757	1.000												
3-FLU	0.639	0.575	0.497	1.000											
1-PYR	0.360	0.449	0.332	0.688	1.000										
2CAEMA	0.483	0.317	0.188	0.579	0.537	1.000									
2CYEMA	0.926	0.864	0.713	0.733	0.473	0.597	1.000								
2COEMA	0.571	0.587	0.569	0.514	0.287	0.337	0.723	1.000							
3HPMA	0.742	0.759	0.739	0.607	0.361	0.460	0.832	0.762	1.000						
MADA	0.577	0.621	0.597	0.791	0.614	0.454	0.716	0.571	0.681	1.000					
PHGA	-0.119	-0.032	-0.002	0.038	0.010	0.203	0.008	0.142	0.144	0.095	1.000				
2HPMA	0.675	0.492	0.526	0.561	0.390	0.477	0.759	0.689	0.750	0.612	0.107	1.000			
t4HBEMA	0.806	0.802	0.704	0.644	0.392	0.388	0.880	0.793	0.906	0.679	0.050	0.721	1.000		
3HMPMA	0.745	0.765	0.724	0.575	0.327	0.318	0.797	0.766	0.937	0.642	0.071	0.714	0.943	1.000	
4HMBEMA	0.780	0.769	0.709	0.646	0.396	0.265	0.829	0.743	0.843	0.655	0.012	0.673	0.919	0.905	1.000
**Abbrev.**	**Compound**				**Parent Compound**		**Abbrev.**		**Compound**				**Parent Compound**		
TNE-2	cotinine + trans-3′-hydroxycotinine				nicotine		3HPMA		N-Acetyl-S-(3-hydroxypropyl)-L-cysteine				acrolein		
NNAL	4-(methylnitrosamino)-1-(3-pyridyl)-1-butanol				NNK		MADA		Mandelic acid				styrene		
NNN	*N’*-nitrosonornicotine				NNN		PHGA		Phenylglyoxylic acid				ethylbenzene and styrene		
3-FLU	3-hydroxyfluorene				fluorene		2HPMA		N-Acetyl-S-(2-hydroxypropyl)-L-cysteine				propylene oxide		
1-PYR	1-hydroxypyrene				pyrene		t4HBEMA		N-Acetyl-S-(4-hydroxy-2-buten-1-yl)-L-cysteine				1,3-butadiene		
2CAEMA	N-Acetyl-S-(2-carbamoylethyl)-L-cysteine				acrylamide		3HMPMA		N-Acetyl-S-(3-hydroxypropyl-1-methyl)-L-cysteine				crotonaldehyde		
2CYEMA	N-Acetyl-S-(2-cyanoethyl)-L-cysteine				Acrylonitrile		4HMBEMA		N-Acetyl-S-(4-hydroxy-2-methyl-2-buten-1-yl)-L-cysteine				isoprene		
2COEMA	N-Acetyl-S-(2-carboxyethyl)-L-cysteine				Acrolein										

**Table 4:** Matched odds ratios for lung cancer incidence by biomarker concentration in the Golestan Cohort Study.

	Matched Odds Ratio (95% CI) (1)	Matched Odds Ratio (95% CI) Adjusted for Demographics (2)	Matched Odds Ratio (95% CI) Adjusted for Demographics and Opium (3)	Matched Odds Ratio (95% CI) Adjusted for Demographics and Smoking (4)	Matched Odds Ratio (95% CI) Adjusted for Demographics, Opium, and Smoking (5)
TNE-2	2.22 (1.03, 4.78)	2.20 (1.01, 4.83)	1.70 (0.79, 3.65)	2.06 (0.96, 4.42)	1.68 (0.89, 3.17)
NNAL	1.46 (0.75, 2.82)	1.44 (0.75, 2.78)	1.01 (0.47, 2.16)	1.35 (0.59, 3.07)	0.72 (0.23, 2.24)
NNN	2.44 (1.13, 5.27)	2.28 (1.04, 5.00)	1.91 (0.85, 4.28)	2.14 (0.81, 5.60)	1.90 (0.63, 5.69)
3-FLU	1.57 (0.90, 2.71)	1.62 (0.92, 2.84)	1.12 (0.58, 2.17)	1.53 (0.87, 2.69)	1.03 (0.53, 2.02)
1-PYR	1.94 (0.93, 4.07)	2.47 (1.06, 5.77)	1.87 (0.71, 4.89)	2.34 (0.99, 5.52)	1.64 (0.60, 4.49)
2CAEMA	2.00 (1.03, 3.88)	2.17 (1.06, 4.44)	1.35 (0.57, 3.22)	2.14 (1.01, 4.55)	1.34 (0.55, 3.28)
2CYEMA	2.17 (1.03, 4.58)	2.10 (1.04, 4.22)	1.66 (0.82, 3.34)	2.17 (1.03, 4.55)	1.79 (0.88, 3.65)
2COEMA	2.16 (0.85, 5.48)	2.20 (0.82, 5.92)	1.86 (0.65, 5.34)	2.02 (0.69, 5.89)	2.83 (0.62, 12.84)
3HPMA	2.19 (1.03, 4.66)	2.34 (1.03, 5.34)	1.72 (0.73, 4.03)	2.27 (0.91, 5.68)	1.94 (0.68, 5.60)
MADA	3.63 (1.00, 13.18)	4.77 (1.13, 20.11)	2.55 (0.54, 12.04)	3.97 (0.77, 20.37)	2.03 (0.33, 12.57)
PHGA	1.41 (0.82, 2.43)	1.52 (0.85, 2.72)	1.22 (0.65, 2.30)	1.45 (0.77, 2.70)	1.14 (0.55, 2.34)
2HPMA	2.72 (1.16, 6.36)	2.79 (1.16, 6.73)	1.88 (0.73, 4.82)	2.85 (1.04, 7.81)	2.08 (0.73, 5.89)
t4HBEMA	1.85 (0.94, 3.62)	1.97 (0.95, 4.10)	1.54 (0.72, 3.29)	1.82 (0.84, 3.93)	1.58 (0.66, 3.79)
3HMPMA	1.70 (0.85, 3.37)	1.81 (0.85, 3.90)	1.40 (0.63, 3.14)	1.73 (0.76, 3.93)	1.47 (0.57, 3.75)
4HMBEMA	1.43 (0.87, 2.34)	1.47 (0.87, 2.47)	1.25 (0.71, 2.19)	1.39 (0.81, 2.36)	1.23 (0.67, 2.25)

Abbreviation: CI = confidence interval. (1) Matched odds ratios were obtained from the conditional logistic regression analyses that accounted for the matching of the cases and controls based on sex, age, urban/rural residence, cohort enrollment time, and duration of the follow-up, and adjusted for log-transformed creatinine values as a covariable. They represent the odds ratios associated with a log-unit increase of the relevant biomarker. (2) Matched odds ratios additionally adjusted for education and ethnicity. (3) Matched odds ratios additionally adjusted for education, ethnicity, and occurrence of regular opium use. (4) Matched odds ratios additionally adjusted for education, ethnicity, cigarettes smoked per day, and years of cigarette smoking. (5) Matched odds ratios additionally adjusted for education, ethnicity, occurrence of regular opium use, cigarettes smoked per day, and years of cigarette smoking.

## Data Availability

The data presented in this study are available upon reasonable request submitted through the study portal: https://dceg2.cancer.gov/gemshare/ (accessed on 27 May 2021).

## Supplementary Materials

The following are available online at https://www.mdpi.com/article/10.3390/ijerph18147349/s1: Figure S1: Flowchart of Sample Inclusion and Exclusion Criteria; Table S1. Geometric mean (with 95% CI) of creatinine-corrected biomarkers concentrations among current exclusive cigarette smokers (*n* = 80) in the Golestan Cohort Study; Table S2. Correlation between biomarker levels among exclusive current cigarette controls in the Golestan Cohort Study (*n* = 52); and Table S3. Risk of lung cancer by biomarkers among current exclusive cigarette users (*n* = 80).
