# Supplementary figures and images for: Associations between Biomarkers of Exposure and Lung Cancer Risk among Exclusive Cigarette Smokers in the Golestan Cohort Study
Source: Int J Environ Res Public Health. 2021 Jul 9;18(14):7349. doi: 10.3390/ijerph18147349 (PMC8306295; doi:10.3390/ijerph18147349)

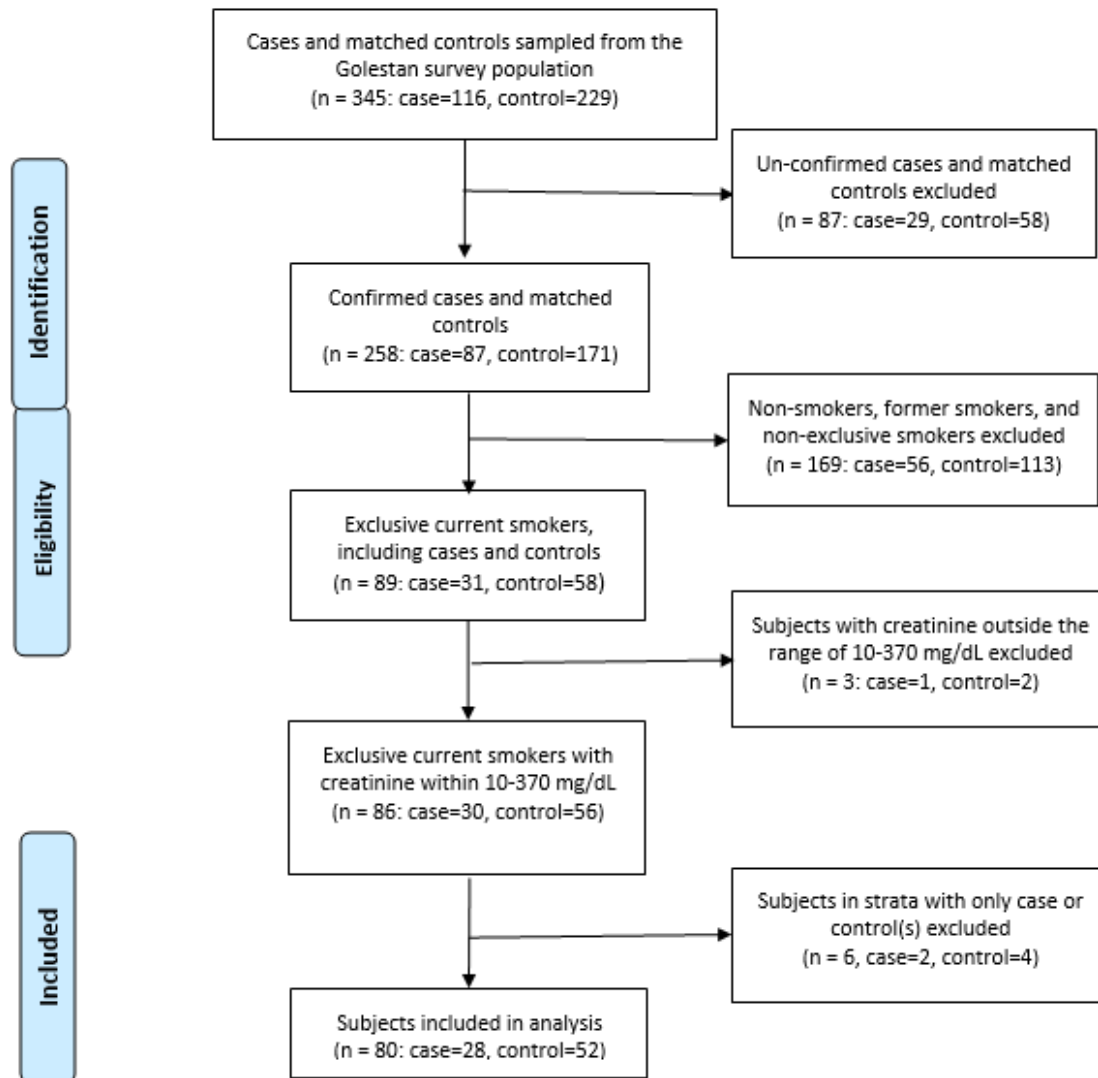

Figure S1. Flowchart of Sample Inclusion and Exclusion Criteria.

Supplement: Supplementary file 1 [file ijerph-18-07349-s001.zip › Golestan Lung Cancer Flow Diagram.pdf]
